# Huntsman spiders of the genus *Sinopoda* (Araneae, Sparassidae, Heteropodinae) from the Honghe Hani and Yi Autonomous Prefecture, southwestern China, with descriptions of two new species

**DOI:** 10.3897/zookeys.1280.183824

**Published:** 2026-05-28

**Authors:** Chengwen Zhang, Yuanqian Xing, Yang Zhong, Hao Yu

**Affiliations:** 1 The State Key Laboratory of Southwest Karst Mountain Biodiversity Conservation of Forestry Administration, School of life sciences, Guizhou Normal University, Guiyang, Guizhou, China Guizhou Normal University Guiyang China; 2 School of Nuclear Technology and Chemistry & Biology, Hubei University of Science and Technology, Xianning, Hubei, China Hubei University of Science and Technology Hubei China

**Keywords:** Biodiversity, COI, DNA barcoding, fauna, morphology, taxonomy, Yunnan Province

## Abstract

Spiders of the genus *Sinopoda* Jäger, 1999 from Honghe Hani and Yi Autonomous Prefecture, Yunnan Province, China are studied. A total of four species are reported and illustrated of which two, *Sinopoda honghe* Yu & Zhong, **sp. nov**. and *Sinopoda kuan* Yu & Zhong, **sp. nov**., are described as new to science. The other two previously described species from this region: *S. tengchongensis* Fu & Zhu, 2008 and *S. tumefacta* Zhong, Jäger, Chen & Liu, 2019 are also illustrated. Detailed descriptions, diagnoses, illustrations and DNA barcodes of the two new species are given. A distribution map of these four species in Honghe is provided.

## Introduction

*Sinopoda* Jäger, 1999 is the fourth most species-rich genus in the huntsman spider family Sparassidae, following *Pseudopoda* Jäger, 2000 (271 species), *Heteropoda* Latreille, 1804 (228 species), and *Olios* Walckenaer, 1837 (169 species) (World Spider Catalog 2026). To date, 141 species of *Sinopoda* have been described, predominantly distributed across eastern Asia (World Spider Catalog 2026). Among these, 91 species occur in East Asia, 50 in Southeast Asia, and one in India (Zhang et al. 2023a, 2024). China harbors the greatest diversity of the genus, with 75 species documented, representing 53.19% of the global total (World Spider Catalog 2026). Most Chinese *Sinopoda* species have been comprehensively studied, particularly those described in recent years, with detailed morphological descriptions and high-quality illustrations that facilitate accurate species identification (Zhang et al. 2015, 2021, 2023a, 2023c, 2024; Zhong et al. 2017, 2018, 2019, 2022; Grall and Jäger 2020; Zhu et al. 2020; Wang et al. 2021). However, the diversity of this genus in China is still insufficiently known, and several new species have been described in the last few years (World Spider Catalog 2026).

**Figure 1.**
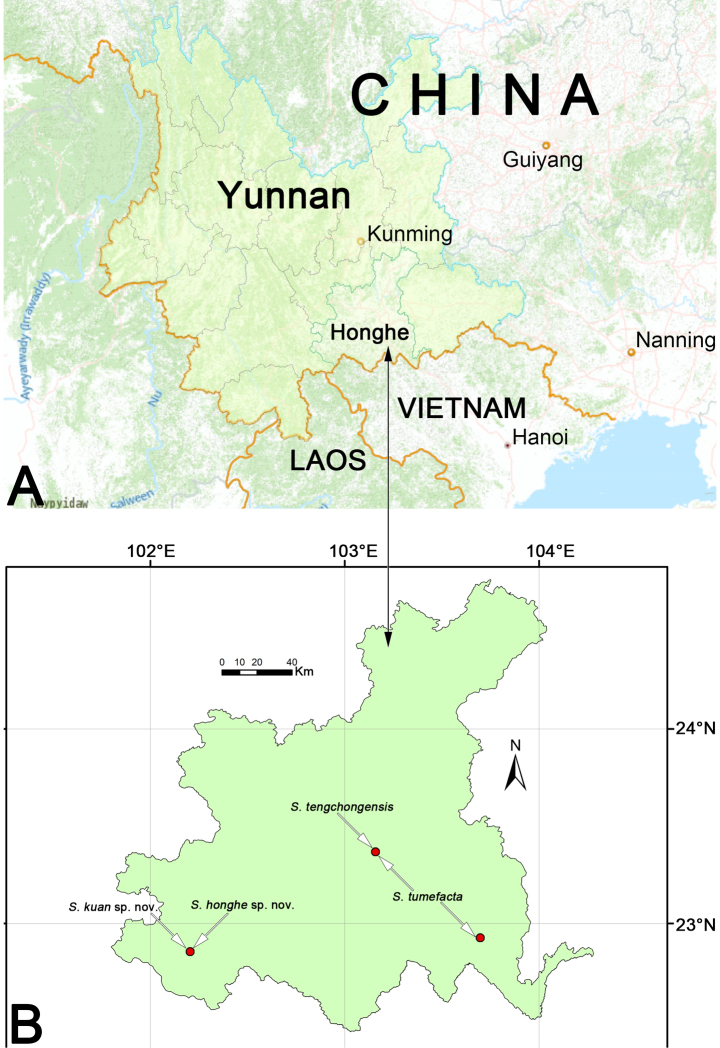
Locality of Honghe Hani and Yi Autonomous Prefecture (**A**) and distribution records (**B**) of *Sinopoda* species in Honghe Prefecture.

The Honghe Hani and Yi Autonomous Prefecture (hereinafter referred to as Honghe Prefecture) is located in the southeastern part of Yunnan Province, China. This province has the richest biodiversity in the country. The prefecture lies at the junction of China and Southeast Asia, bordering Vietnam and Laos, two countries known for their rich biodiversity (Fig. 1). The Honghe Prefecture, with the highest altitude of 3074 m, has a forest coverage of 57.8%, with forest types gradually transitioning from tropical to warm temperate (Lin et al. 2022). It is one of China’s three major biodiversity conservation centers and is recognized as one of the world’s most biologically diverse ‘gree n deltas’ (Zhang et al. 2023b).

Based on the known distribution patterns of *Sinopoda*, Zhang et al. (2024) reported that species of this genus exhibit a strong preference for humid, warm, high-altitude environments and are largely associated with mountainous forest ecosystems, which coincides well with the environmental conditions of Honghe Prefecture. However, there are few records of *Sinopoda* reported from the region, with only two species formally recorded by colleagues from the Centre for Behavioral Ecology and Evolution (CBEE) of Hubei University: *S. tengchongensis* Fu & Zhu, 2008 (widely distributed in Southeast Asia) and *S. tumefacta* Zhong, Jäger, Chen & Liu, 2019 (endemic to Honghe) (Table 1). Notably, *S. tengchongensis* was originally described by Fu and Zhu (2008) and subsequently redescribed by Grall and Jäger (2020); however, both studies provided only line drawings of the genitalia without any microphotographs. For *S. tumefacta*, although microphotographs of the genitalia have been presented in several papers (Zhong et al. 2019; Zhang et al. 2021, 2024), they are not assembled in a single plate, and none of the key structures (e.g., Em—embolus, RTA, LL—lateral lobes) are labelled. This inevitably causes inconvenience for species identification and reference.

**Table 1.** Checklist of *Sinopoda* species from Honghe Hani and Yi Autonomous Prefecture.

	Species name	Authorship	Known sex	Distribution	References
1	*S. tengchongensis*	Fu & Zhu, 2008	♂♀	Thailand [Phrae]; China [Yunnan (Honghe Pref., Dali Pref., Nujiang Pref.), Hainan, Hunan]	Zhang et al. 2024; photomicrographs supplemented in present paper
2	*S. tumefacta*	Zhong, Jäger, Chen & Liu, 2019	♂♀	Endemic to Honghe Pref.	Zhong et al. 2019; Zhang et al. 2024; present paper
3	*S. honghe*	Yu & Zhong, sp. nov.	♂♀	Endemic to Honghe Pref.	present paper
4	*S. kuan*	Yu & Zhong, sp. nov.	♀	Endemic to Honghe Pref.	present paper

Field collection in Honghe Prefecture was carried out in April, 2024 (Fig. 1). During this field exploration, we collected spider specimens of the genus *Sinopoda*. Based on morphological and molecular evidence, the specimens were identified as two undescribed species.

The aim of the current paper is to present all species of *Sinopoda* spiders currently known from Honghe Prefecture, including: 1) descriptions of *S. honghe* Yu & Zhong, sp. nov. and *S. kuan* Yu & Zhong, sp. nov., with the COI sequence DNA barcodes; 2) re-illustration of *S. tengchongensis* and *S. tumefacta* based on collections of CBEE, including supplementary micrographs of *S. tengchongensis*; and 3) a distribution map of *Sinopoda* species in Honghe Prefecture.

## Material and methods

Specimens in this study were collected by hand. The type specimens of the two new species are deposited in the Museum of Guizhou Normal University (**MGNU**), Guiyang, China, while the material examined of the two known species is deposited in the Centre for Behavioral Ecology and Evolution of Hubei University (**CBEE**), Wuhan, China.

Specimens were examined using an Olympus SZX7 stereomicroscope. Further details were studied under a CX41 compound microscope. Left male palps were examined and illustrated after dissection. Epigynes were removed and cleared in a warm 10% potassium hydroxide (KOH) solution. Images were captured with a Canon EOS 70D digital camera mounted on an Olympus CX41 compound microscope and assembled using Helicon Focus 6.80 image stacking software (Khmelik et al. 2005). All measurements were obtained using an Olympus SZX7 stereomicroscope and are given in millimeters. Eye diameters were measured at the widest part. The total body length does not include the chelicerae or spinnerets. Leg lengths are given as total length (femur, patella + tibia, metatarsus, tarsus). The number of macrosetae is listed for each segment in the following order: prolateral, dorsal, retrolateral, and ventral (in femora and patellae, ventral spines are absent, and the fourth digit is omitted in the setation formula). The ratio between palpal tibia length and cymbium was calculated by measuring both parts in a retrolateral view and dividing the value of the tibia length by the cymbial length value. The same method was used to calculate the ratios of the dRTA to the palpal tibia and the vRTA to the dRTA.

The distribution map was generated with ArcGIS 10.5 (ESRI Inc 2002). The terminology used in the text and figure legends follows Grall and Jäger (2020), Zhong et al. (2017, 2018, 2019), and Zhang et al. (2021, 2023a, 2024). Abbreviations used in the text and figures are given in Table 2.

**Table 2.** List of abbreviations used in the text and figures.

**Male palp**	
**C** = conductor	**CB** = cymbial bulge
**Cy** = cymbium	**dRTA** = dorsal branch of RTA
**EA** = embolic apophysis	**EB** = embolic base
**Em** = embolus	**ET** = embolic tip
**Sp** = spermophor	**St** = subtegulum
**T** = tegulum	**Ti =** palpal tibia
**vRTA** = ventral branch of RTA	**RTA** = retrolateral tibial apophysis
**Epigyne**	
**A** = atrium	**AB** = anterior band
**amEF** = anterior margin of epigynal field	**amLL** = anterior margin of lateral lobes
**FB** = fusion bubbles	**FD** = fertilization duct
**GA** = glandular appendages	**ID** = internal ducts
**LL** = lateral lobes	**LS** = lobal septum
**MS** = membranous sac	**pmLL** = posterior margin of lateral lobes
**PP** = posterior part of spermathecae	**SB** = sclerotised bulges
**Ocular area**	
**ALE** = anterior lateral eye	**ALE–PLE** = distance between ALE and PLE
**AME** = anterior median eye	**AME–ALE** = distance between AME and ALE
**AME–AME** = distance between AMEs	**AME–PME** = distance between AME and PME
**PLE** = posterior lateral eye	**PME** = posterior median eye
**PME–PLE** = distance between PME and PLE	**PME–PME** = distance between PMEs
**CH** = clypeus height	
**Legs**	
**Fe** = femur	**Mt** = metatarsus
**Pa** = patella	**Ti** = tibia
**I, II, III, IV** = legs I to IV	

A partial fragment of the mitochondrial gene cytochrome *c* oxidase subunit I (COI) was amplified and sequenced to estimate genetic distances between the two sympatric new species and to confirm identifications and the accuracy of sex pairing. Total genomic DNA was extracted using the Cell & Tissue Genomic DNA Isolation Kit (Bioteke, Beijing, China), following the manufacturer’s protocols. COI was amplified using the primer pairs C1-J-1718 (5’-GGAGGATTTGGAAATTGATTAGTTCC-3’) (Simon et al. 1994) and C1-N-2776 (5’-GGATAATCAGAATATCGTCGAGG-3’) (Hedin and Maddison 2001) with the following PCR reaction protocol: initial denaturation at 94 °C for 5 min; 35 cycles of denaturation at 94 °C for 30 s, annealing at 45 °C for 40 s, and elongation at 72 °C for 1 min; and final extension at 72 °C for 7 min. The 50 μL PCR reactions contained 19.5 μL of double-distilled H_2_O, 25 μL of 2× Taq PCR (MasterMixII TianGen Biotech, Beijing, China), 2 μL of each forward and reverse 10-μM primer, and 1.5 μL of DNA template. The double-stranded PCR products were visualized by agarose gel electrophoresis (1.5% agarose). PCR products were transported to the Beijing Tsingke Biotech Co., Ltd. (Chong Qing, China) to be sequenced using the same PCR primers. We manually edited the sequences using Geneious Prime 2024 (Kearse et al. 2012), and translated nucleotide reads into amino acid sequences to check for stop codons and ensure the proper configuration of codon positions.

Sequences were trimmed to 1001 bp. All sequences were confirmed using BLAST and are deposited in GenBank. The codes and GenBank accession numbers of voucher specimens are provided as follows: *S. honghe* sp. nov.: SZY003, ♂, GenBank PZ339696; SZY004, ♀, GenBank PZ339698; SZY005, ♀, GenBank PZ339697. *S. kuan* sp. nov.: SZY002, ♀, GenBank PZ339695.

## Taxonomic accounts

### Family Sparassidae Bertkau, 1872

**Subfamily Heteropodinae Thorell, 1873**

#### Genus Sinopoda

Taxon classificationAnimaliaAraneaeSparassidae

Jäger, 1999

4862F2C7-5B2A-5EF9-835F-FA9F8F6D3B0F

##### Type species.

*Sarotes forcipatus* (Karsch, 1881) from China and Japan.

##### Diagnosis.

See Jäger (1999), Liu et al. (2008), Zhang et al. (2015) and Grall and Jäger (2020).

##### Distribution.

Widely distributed in East Asia (75 species in China, 16 species in Japan and Korea), Southeast Asia (50 species in Brunei, Indonesia, Laos, Malaysia, Myanmar, Thailand and Vietnam), and South Asia (1 species in India). Also see fig. 1B, C in Zhang et al. (2021).

#### Sinopoda honghe

Taxon classificationAnimaliaAraneaeSparassidae

Yu & Zhong sp. nov.

7CD58081-0389-5B58-BE03-B88A65D8D08A

https://zoobank.org/80C056B4-BD11-466B-8D1A-EE6B61FADD77

Figs 1, 2, 3, 4, 5

##### Material examined.

***Holotype*** ♂ (SZY003) (MGNU), China: • Yunnan Pro.: Honghe Hani and Yi Autonomous Prefecture, Luechun Co., Huanglianshan Mt., 22.85°N, 102.20°E, c. 2637 m, by hand, 16 IV 2024, Y. Zhong & S. Yang leg. ***Paratypes***: 2♀ (SZY004, SZY005) (MGNU), same data as holotype.

##### Etymology.

The specific epithet is derived from the name of the type locality; noun in apposition.

##### Diagnosis.

**Figure 2.**
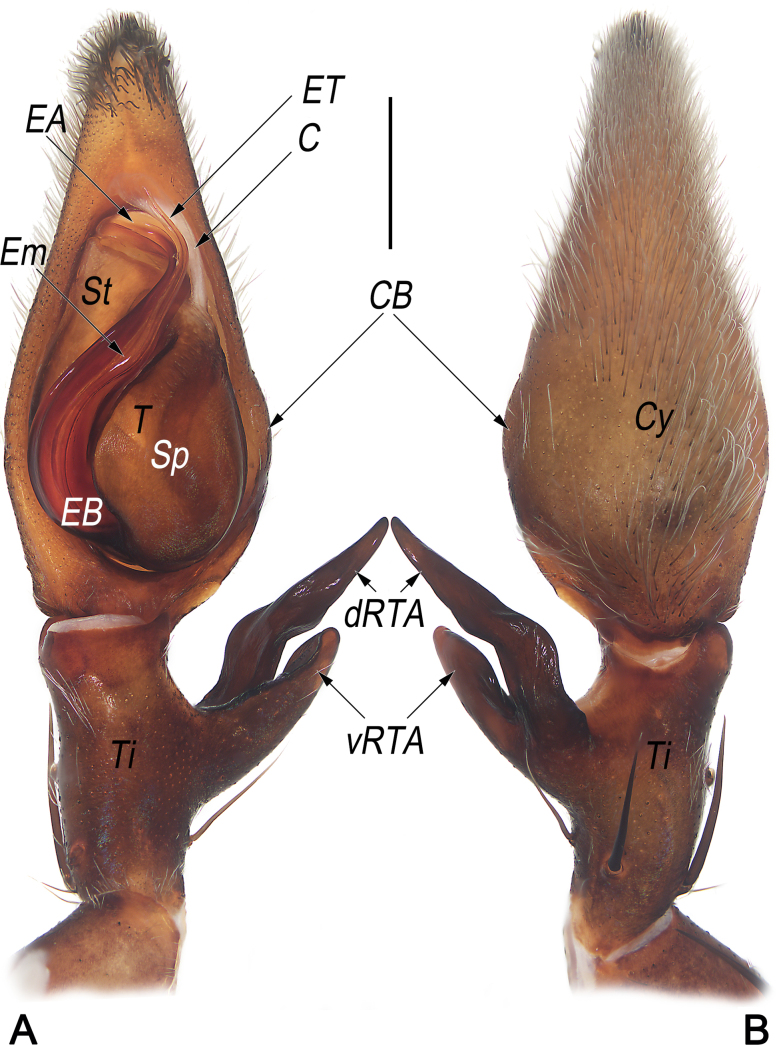
Male palp of the holotype of *Sinopoda honghe* sp. nov. **A**. Ventral; **B**. Dorsal. Abbreviations: C = conductor; CB = cymbial bulge; Cy = cymbium; dRTA = dorsal branch of RTA; EA = embolic apophysis; EB = embolic base; Em = embolus; ET = embolic tip; Sp = spermophor; St = subtegulum; T = tegulum; Ti = palpal tibia; vRTA = ventral branch of RTA. Scale bar: 1 mm (**A, B**).

**Figure 3.**
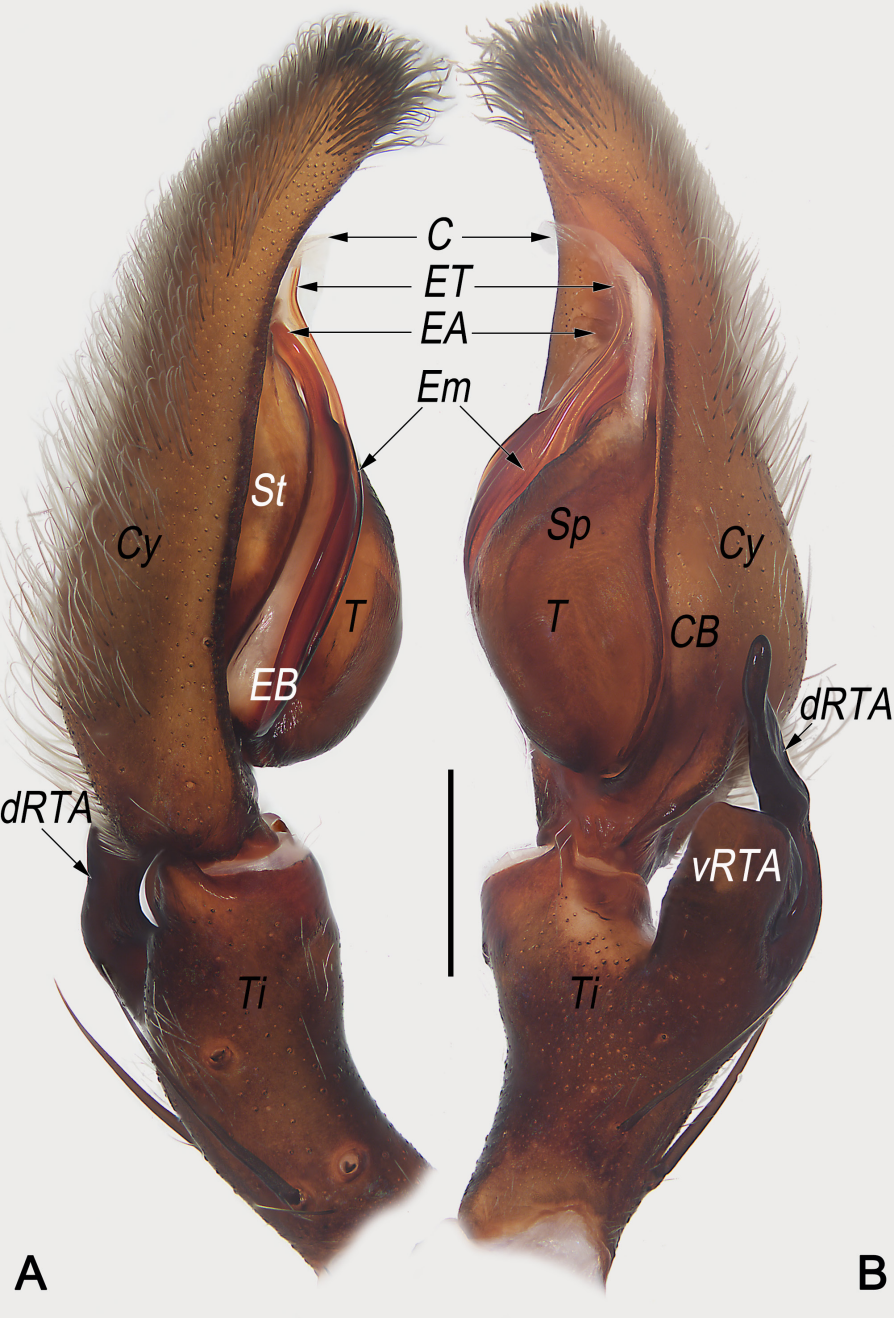
Male palp of the holotype of *Sinopoda honghe* sp. nov. **A**. Prolateral; **B**. Retrolateral. Abbreviations: C = conductor; CB = cymbial bulge; Cy = cymbium; dRTA = dorsal branch of RTA; EA = embolic apophysis; EB = embolic base; Em = embolus; ET = embolic tip; Sp = spermophor; St = subtegulum; T = tegulum; Ti = palpal tibia; vRTA = ventral branch of RTA. Scale bar: 1 mm (**A, B**).

The male of *S. honghe* sp. nov. resembles that of *S. pengi* Song & Zhu, 1999 in the general shape of the male palp. The palps of the two species share the similarly shaped conductor (C), embolus (Em), embolic apophysis (EA) and the distinctly long, finger-shaped dorsal branch of the RTA (dRTA) which has lumpy margins but differs in the following: (1) apex of dRTA blunt (vs. sharp) (cf. Figs 2A, 2B, 3B and Zhong et al. 2019: figs 46B, C, 47B–D, 48B–D); (2) ventral margin of branch of RTA (vRTA) smooth, without triangular apophysis (vRTA with a ventral triangular apophysis) (cf. Figs 2A, 3B and Zhong et al. 2019: figs 46C, 47C, D, 48C, D). The female of *S. honghe* sp. nov. is similar to that of *S. tham* Jäger, 2012 in having narrow, largely transversally oriented lateral lobes (LL) and epigynal pockets with a narrow lobal septum (LS) as well as an internal duct system (ID) with a long and narrow parallel part but can be recognised by: (1) anterior keel of lobal septum (LS) distinctly narrow, about 1/12 width of the epigynal plate (vs. relatively wider, about 1/8 width of the epigynal plate) (cf. Fig. 5A, B and Jäger 2012: figs 18, 22); (2) glandular appendages (GA) tubular, distally abruptly bent and directed posteriorly (vs. distally not bent, just slightly curved, apex directed posterolaterally) (cf. Fig. 5C and Jäger 2012: figs 19, 20, 23).

##### Description.

**Male (SZY003)**. Total length 16.2. Carapace 7.6 long, 6.8 wide, anterior width 4.1. Opisthosoma 8.6 long, 4.5 wide. ***Eye sizes and interdistances***: AME 0.36, ALE 0.57, PME 0.41, PLE 0.53, AME–AME 0.25, AME–ALE 0.06, PME–PME 0.38, PME–PLE 0.54, AME–PME 0.59, ALE–PLE 0.50, CHAME 0.36, CHALE 0.37. ***Spination***: palp: 131, 101, 1211; Fe: I–III 323, IV 321; Pa: I–IV 101; Ti: I–II 2126, III–IV 3136; Mt: I–II 2024, III–IV 3036. ***Measurements of palp and legs***: palp 12.5 (4.4, 1.8, 2.3, 4.0), I 39.3 (10.4, 3.8, 10.6, 11.0, 3.5), II 43.1 (11.7, 3.9, 11.9, 11.9, 3.7), III 34.6 (9.9, 3.6, 9.2, 9.1, 2.8), IV 36.4 (9.9, 3.3, 9.8, 10.2, 3.2). Cheliceral furrow with three anterior and four posterior teeth, and with ~50 denticles.

**Figure 4.**
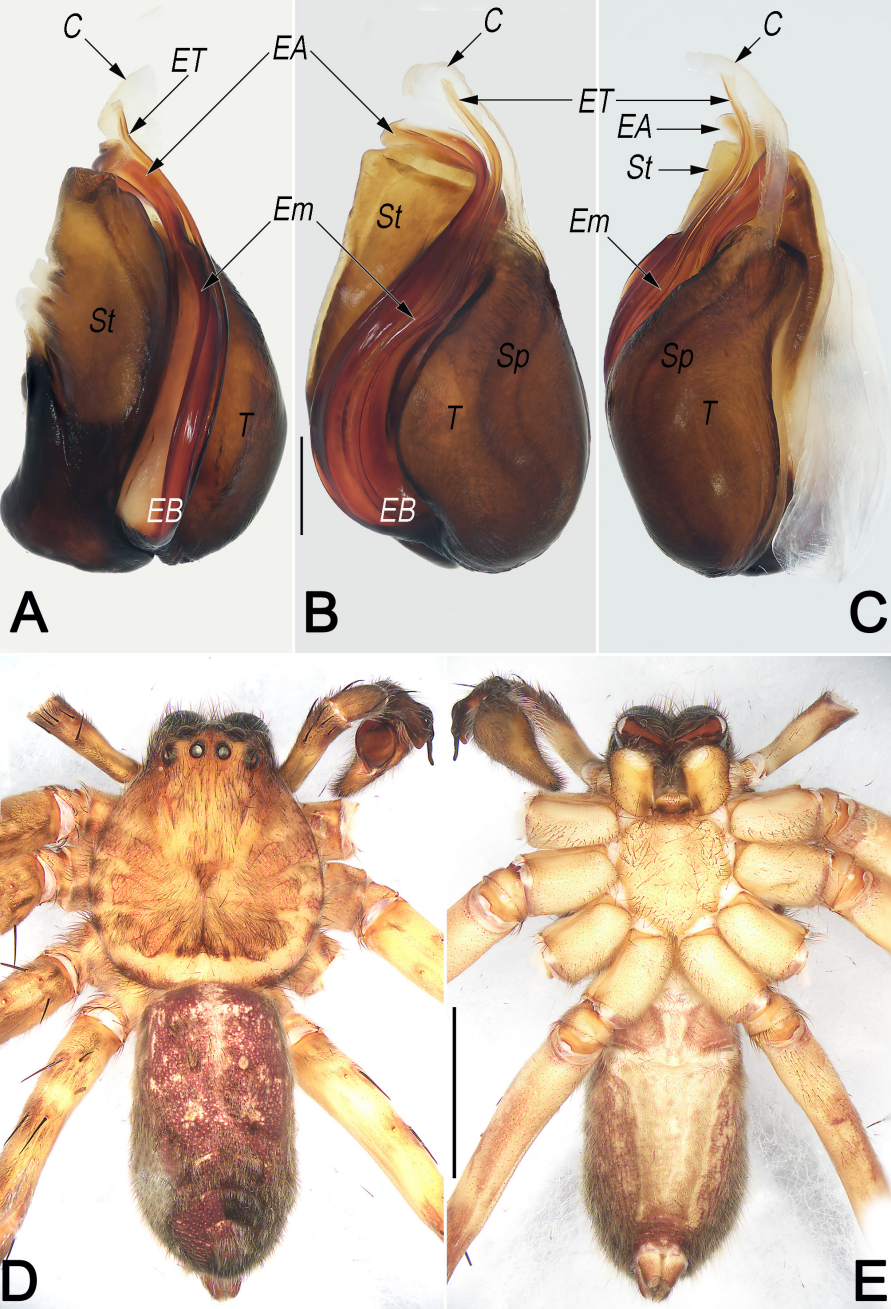
*Sinopoda honghe* sp. nov., male holotype, palpal bulb (**A–C**) and habitus (**D, E**). **A**. Prolateral; **B, E**. Ventral; **C**. Retrolateral; **D**. Dorsal. Abbreviations: C = conductor; EA = embolic apophysis; EB = embolic base; Em = embolus; ET = embolic tip; Sp = spermophor; St = subtegulum; T = tegulum. Scale bars: 0.5 mm (**A–C**); 5 mm (**D, E**).

***Colouration in ethanol* (Fig. 4D, E)**. Carapace deep yellowish to brown, with yellow submarginal transversal band posteriorly. Median band anteriorly wide, posteriorly narrowed, bright yellowish brown, lateral bands brown and not distinctly delimited to median band; cervical groove and radial grooves distinct. Sternum yellowish white. Opisthosoma dorsally reddish brown, with a nearly T-shaped bright yellow median band, reaching 1/2 of abdomen length, with two pairs of inconspicuous black dots on each side, posteriorly with three transverse chevrons; ventral opisthosoma yellowish white, uniformly coloured and lacking markings.

**Figure 5.**
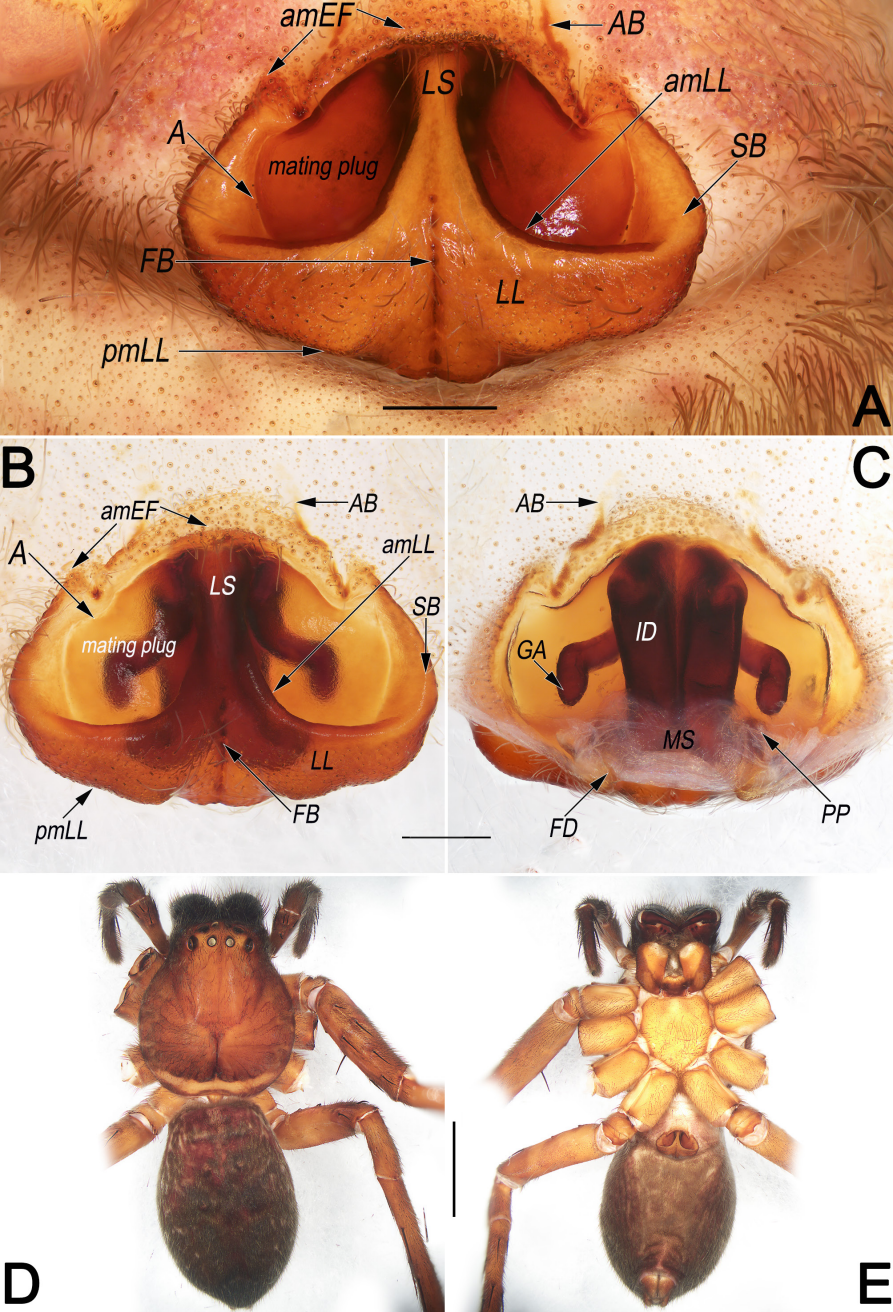
*Sinopoda honghe* sp. nov., female paratype, epigyne (**A–C**) and habitus (**D, E**). **A**. Intact, ventral; **B**. Cleared and macerated, ventral; **C**. Cleared and macerated, dorsal; **D**. Dorsal; **E**. Ventral. Abbreviations: A = atrium; AB = anterior band; amEF = anterior margin of epigynal field; amLL = anterior margin of lateral lobes; FB = fusion bubbles; FD = fertilization duct; GA = glandular appendages; ID = internal ducts; LL = lateral lobes; LS = lobal septum; MS = membranous sac; pmLL = posterior margin of lateral lobes; PP = posterior part of spermathecae; SB = sclerotised bulges. Scale bars: 0.5 mm (**A–C**); 5 mm (**D, E**).

***Palp* (Figs 2, 3, 4A–C)**. Tibia (Ti) moderately long, c. 1/2 length of cymbium, with retrolateral apophysis (RTA) arising medially; ventral branch of RTA (vRTA) relatively short, c. 1/2 length of dRTA, shovel-shaped in ventral view, nearly rectangular and rounded at corners in retrolateral view; dorsal branch of RTA (dRTA) nearly finger-shaped, margins undulating, tapering toward tip, approximately as long as tibia, extending to cymbial base. Cymbium (Cy) slender, c. 2.6× longer than wide, cymbial bulge (CB) indistinct. Tegulum (T) elongate-oval, proximally strongly bulged and prolapsed, distinctly excavated on prolatero-apical side to accommodate embolus (Em) and conductor (C); spermophor (Sp) distinct, ‘(’-shaped in ventral view. Embolus (Em) slightly curved, nearly Ƨ-shaped in ventral view, at least 1.5× longer than tegulum length; the embolic base (EB) distinctly thick, arising at approximately the 8–9 o’clock position; the free part of the embolus (Em) filiform; the embolic tip (ET) curved and apically sharp, terminating at c. 12 o’clock position. Embolic apophysis (EA) ribbon-like, nearly as long as embolus, proximally extending alongside embolus, distally bent at right angle and separating from embolus, apex blunt and directed prolaterodistally. Conductor (C) membranous, c. 1/2 of embolus length, originating on retrolateral flank (c. 1–2 o’clock on tegulum), terminating at c. 11–12 o’clock position; conductor (C) proximally narrowed, medially slightly curved, tip widened, shaped like the membranous wing of hymenoptera, directed prolaterodistally and apically beyond embolic tip (ET).

**Female (SZY004)**. Total length 20.3. Carapace 9.1 long, 8.0 wide, anterior width 5.2. Opisthosoma 11.2 long, 7.1 wide. ***Eye sizes and interdistances***: AME 0.47, ALE 0.63, PME 0.48, PLE 0.60, AME–AME 0.33, AME–ALE 0.12, PME–PME 0.49, PME–PLE 0.62, AME–PME 0.65, ALE–PLE 0.59, CHAME 0.61, CHALE 0.67. ***Spination***: palp: 131, 101, 2121, 1014; Fe: I–III 323, IV 321; Pa: I–IV 101; Ti: I–II 1018, III–IV 2026; Mt: I–II 1014, III–IV 3036. ***Measurements of palp and legs***: palp 13.1 (4.0, 2.1, 2.8, 4.2), I 33.0 (9.2, 4.4, 8.8, 8.0, 2.6), II 36.2 (10.1, 4.6, 9.2, 9.4, 2.9), III 30.6 (9.0, 4.0, 8.0, 7.2, 2.4), IV 31.8 (9.2, 3.8, 7.9, 8.2, 2.7). Cheliceral furrow with three anterior and four posterior teeth, and with ~56 denticles. Colouration in ethanol as in males, but body distinctly darker (Fig. 5D, E).

***Epigyne* (Fig. 5A–C)**. Epigynal field c. 1.5× wider than long; anterior margin of epigynal field (amEF) distinct, trilobate, medially distinctly recurved and arc-shaped, anterolaterally with two small, v-shaped incisions that are separated by c. 6.5× widths; anterior bands (AB) linear, reddish brown, long and distinct, situated at the two incisions. Atrium (A) represented by two symmetrical, reniform cavities divided by the lobal septum (LS), blocked by mating plug, medially and posteriorly delimited by anterior margins of lateral lobes (amLL), laterally bordered by sclerotised bulges (SB). Lobal septum (LS) moderately wide, with anterior keel about 1/12 width of epigynal plate, gradually widening posteriorly. Lateral lobes (LL) nearly boomerang-shaped, fused along a fovea on the central axis; fovea nearly as long as lobal septum, bearing distinct fusion bubbles (FB) aligned along its length; anterior margins of lateral lobes (amLL) distinctly procurved and clearly delimited, semicircular; posterior margins of lateral lobes (pmLL) slightly procurved and bilobed, with a small median incision. Internal ducts (ID) with diameter approximately 1/7 width of epigynal plate, running parallel along median line, nearly as long as epigyne length, slightly convergent posteriorly. Glandular appendages (GA) tubular, of uniform thickness, proximally slightly curved and descending obliquely, nearly ‘(’-shaped and partly covered by internal ducts in dorsal view, distally abruptly bent and directed posteriorly. Posterior part of spermathecae (PP) papilliform, distinctly small, completely obscured by membranous sac (MS) in dorsal view, widely separated by at least 7× diameters. Fertilization ducts (FD) acicular, membranous, originating from the dorsal-basal surface of spermathecae. Membranous sac (MS) between fertilization ducts, nearly disc-shaped.

##### Distribution.

Known only from the type locality (Fig. 1).

#### Sinopoda kuan

Taxon classificationAnimaliaAraneaeSparassidae

Yu & Zhong sp. nov.

0A843D45-0AC3-5287-AF73-87F8F3104EDD

https://zoobank.org/FF2BD52D-6798-4992-99E9-52708FFE60E4

Figs 1, 6

##### Material examined.

***Holotype*** ♀ (SZY002) (MGNU), China: • Yunnan Pro.: Honghe Hani and Yi Autonomous Prefecture, Luechun Co., Huanglianshan Mt., 22.85°N, 102.20°E, c. 2637 m, by hand, 16 IV 2024, Y. Zhong & S. Yang leg.

##### Etymology.

The specific name is derived from the Chinese pinyin ‘kuān’, which means ‘wide’, referring to the distinctly wide lobal septum; adjective.

##### Diagnosis.

**Figure 6.**
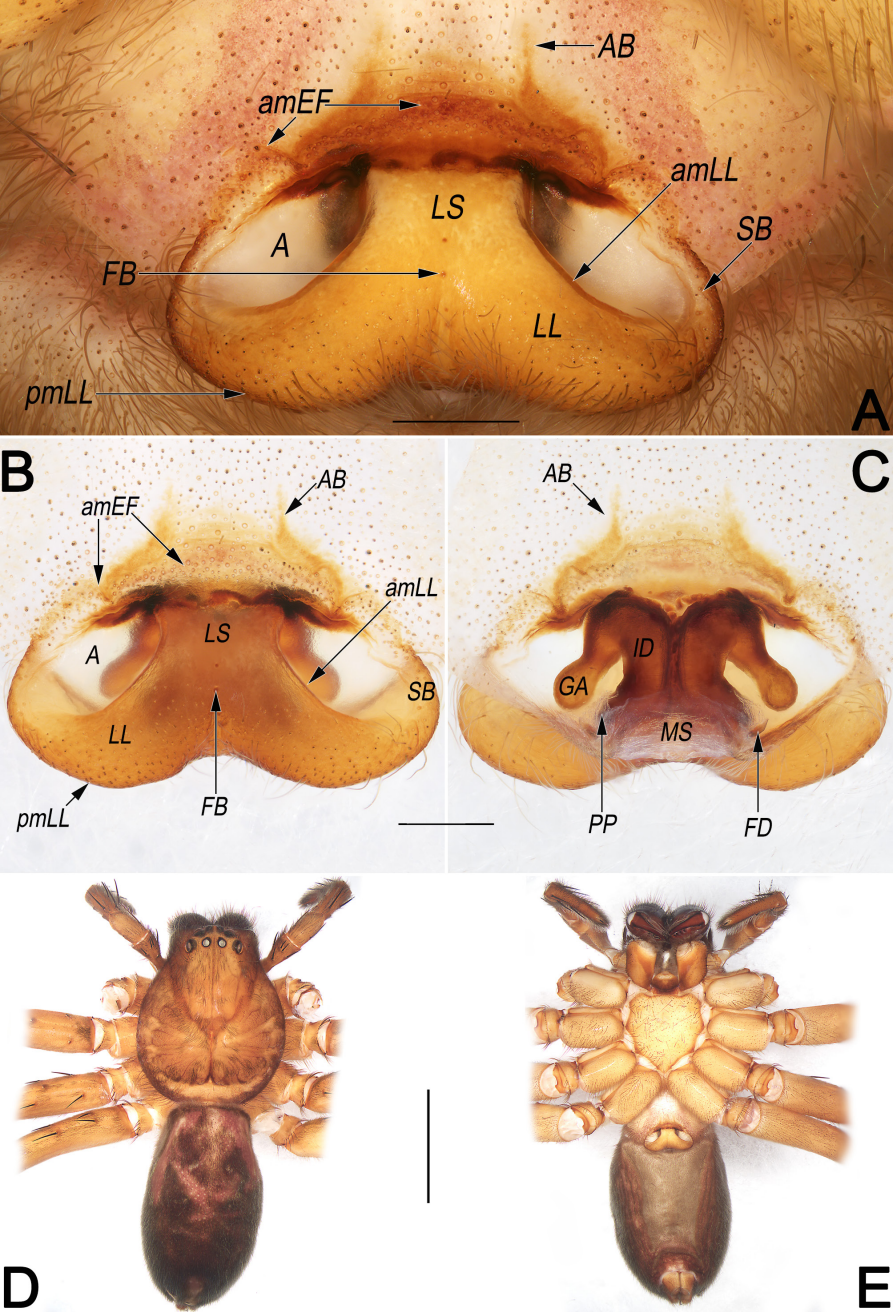
*Sinopoda kuan* sp. nov., female holotype, epigyne (**A–C**) and habitus (**D, E**). **A**. Intact, ventral; **B**. Cleared and macerated, ventral; **C**. Cleared and macerated, dorsal; **D**. Dorsal; **E**. Ventral. Abbreviations: A = atrium; AB = anterior band; amEF = anterior margin of epigynal field; amLL = anterior margin of lateral lobes; FB = fusion bubbles; FD = fertilization duct; GA = glandular appendages; ID = internal ducts; LL = lateral lobes; LS = lobal septum; MS = membranous sac; pmLL = posterior margin of lateral lobes; PP = posterior part of spermathecae; SB = sclerotised bulges. Scale bars: 0.5 mm (**A–C**); 5 mm (**D, E**).

The female of this new species is easily differentiated from other members of the tumefacta group by having a wide lobal septum (LS) that is about 1/3 the width of the epigynal plate, nearly as wide as atrial cavities (vs. no more than 1/5 width of epigynal plate, distinctly narrower than atrial cavities in all other species of the tumefacta group) (cf. Fig. 6A, B and Zhang et al. 2024: S13, fig. A13A, C, E, G, I, K), and by medially narrowed, peanut-shaped glandular appendages (GA) (vs. GA of almost all other *S. tumefacta*-group species medially not narrowed; thumb-like in *S. crassa*, *S. dehiscens*, *S. erromena* and *S. yanlingensis* and finger-shaped in *S. tumefacta* and *S. yaojingensis*) (cf. Fig. 6C and Zhang et al. 2024: S13, fig. A13B, D, F, H, J, L).

##### Description.

**Female (SZY002)**. Total length 16.7. Carapace 7.7 long, 6.6 wide, anterior width 4.1. Opisthosoma 9.0 long, 5.2 wide. ***Eye sizes and interdistances***: AME 0.36, ALE 0.51, PME 0.38, PLE 0.47, AME–AME 0.27, AME–ALE 0.08, PME–PME 0.35, PME–PLE 0.44, AME–PME 0.49, ALE–PLE 0.45, CHAME 0.56, CHALE 0.57. ***Spination***: palp: 131, 101, 2121, 1014; Fe: I–III 323, IV 321; Pa: I–IV 101; Ti: I–II 1014, III–IV 2026; Mt: I–II 1014, III 2124, IV 3036. ***Measurements of palp and legs***: palp 11.4 (3,5, 1.7, 2.5, 3.7), I 30.3 (8.7, 3.4, 8.1, 7.6, 2.5), II 33.0 (9.3, 3.9, 8.9, 8.3, 2.6), III 28.1 (8.2, 3.4, 7.4, 6.9, 2.2), IV 31.2 (9.0, 3.3, 7.8, 8.5, 2.6). Cheliceral furrow with three anterior and four posterior teeth, and with ~58 denticles.

***Colouration in ethanol* (Fig. 6D, E)**. Carapace light to deep brown, centrally with Ψ-shaped markings behind posterior eyes, markings starting from behind PME and PLE almost reaching dark fovea, posteriorly with a light brown and ‘‿’-shaped submarginal transversal band; cervical groove and radial grooves distinct. Sternum bright yellow. Opisthosoma dorsally clothed with dense black setae, interspersed with irregular patches of reddish-brown integument; ventral opisthosoma grayish, uniformly coloured and lacking markings.

***Epigyne* (Fig. 6A–C)**. Epigynal field c. 1.75× wider than long; anterior margin of epigynal field (amEF) distinct, trilobate, medially slightly recurved-shaped like a symbol ‘⏜’, anterolaterally with two small v-shaped incisions that are separated by c. 8× widths; anterior bands (AB) reddish brown, originating at incisions, ascending and curved obliquely, more or less ‘╰╮’-shaped. Atrium (A) represented by two symmetrical, fusiform cavities divided by the lobal septum (LS), medially and posteriorly delimited by anterior margins of lateral lobes (amLL), laterally bordered by sclerotised bulges (SB). Lobal septum (LS) relatively wide, with the anterior keel about 1/3 width of epigynal plate (nearly as wide as atrial cavities), gradually widening posteriorly. Lateral lobes (LL) fused along a fovea on the central axis; lobal fovea nearly 2/3 length of epigynal plate, bearing three to four fusion bubbles (FB) aligned along its length; anterior margins of lateral lobes (amLL) distinctly procurved and clearly delimited, arc-shaped; posterior margins of lateral lobes (pmLL) slightly procurved and bilobed, with a small median incision. Internal ducts (ID) with diameter approximately 1/10 width of epigynal plate, running parallel along median line, diverging distinctly anteriorly and posteriorly. Glandular appendages (GA) relatively long, c. 4/5 length of internal ducts (ID), slightly swollen proximally and distally, narrowed medially, shaped like a peanut, descending obliquely, distally directed posterolaterally. Posterior parts of spermathecae (PP) slightly swollen, partly obscured by membranous sac (MS) in dorsal view, separated by c. 2× diameters. Fertilization ducts (FD) acicular and curved, arising postero-laterally from spermathecae. Membranous sac (MS) between fertilization ducts, nearly disc-shaped.

##### Distribution.

Known only from the type locality (Fig. 1).

#### Sinopoda tengchongensis

Taxon classificationAnimaliaAraneaeSparassidae

Fu & Zhu, 2008

7A6DB3FF-54F4-5850-90A4-034A85F5BB01

Figs 1, 7, 8

Sinopoda tengchongensis Fu & Zhu, 2008: 63, figs 1–5 (♂♀); Grall and Jäger 2020: 66, figs 43a–c, 65g–h (♂).

##### Material examined.

1♂, 1♀ (CBEE), China: • Yunnan Pro.: Honghe Hani and Yi Autonomous Prefecture, Gejiu City, Laoyinshan Mt., 23.36°N, 103.36°E, c. 1955 m, by hand, 29 X 2015, Y. Zhong & Y. Zhu leg.

##### Diagnosis and description.

**Figure 7.**
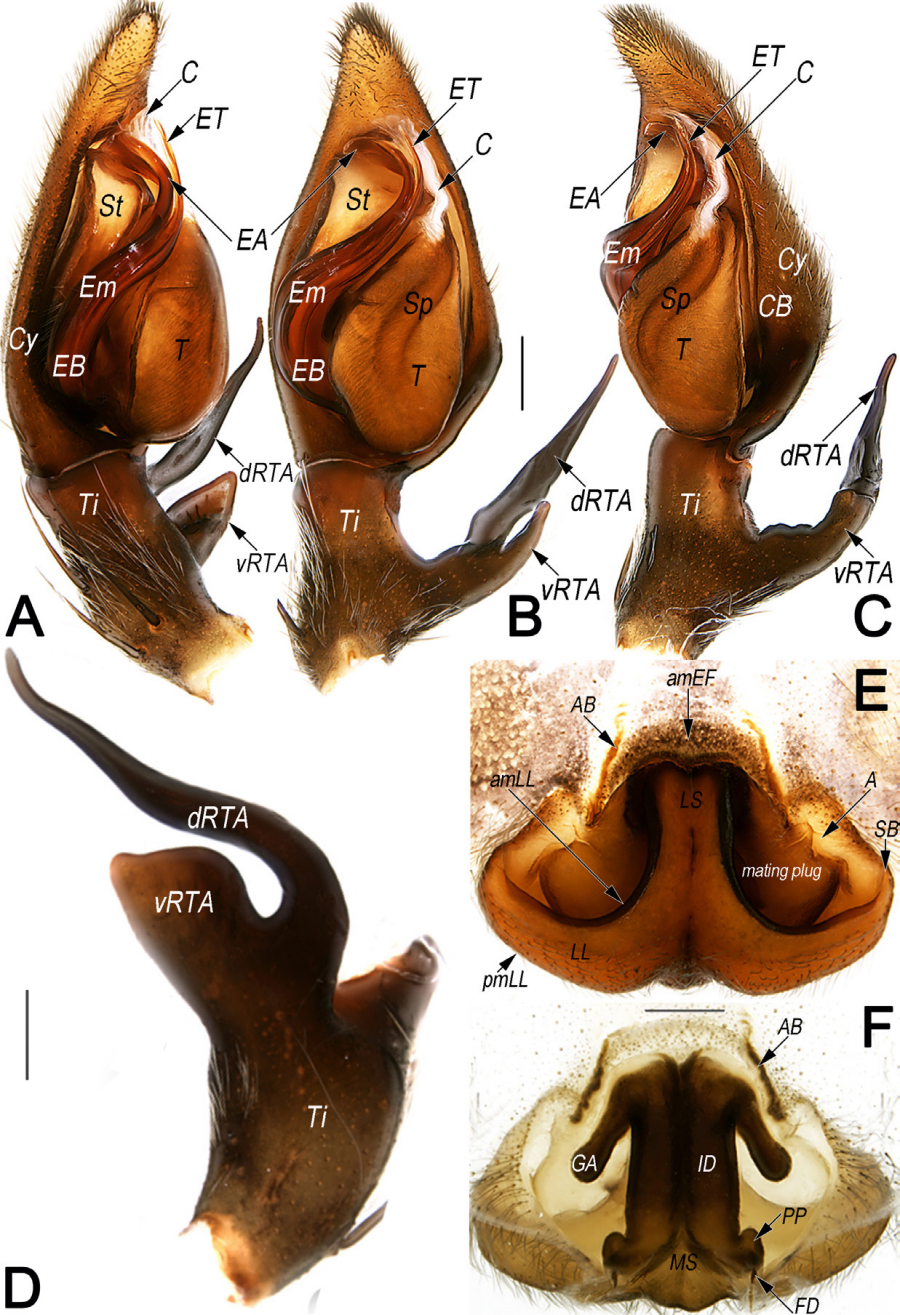
*Sinopoda tengchongensis*, materials from Honghe, male palp (**A–C**), palpal tibia (**D**) and epigyne (**E, F**). **A**. Prolateral; **B, E**. Ventral; **C**. Retrolateral; **D**. Dorsoretrolateral; **F**. Dorsal. Abbreviations: A = atrium; AB = anterior band; amEF = anterior margin of epigynal field; amLL = anterior margin of lateral lobes; C = conductor; CB = cymbial bulge; Cy = cymbium; dRTA = dorsal branch of RTA; EA = embolic apophysis; EB = embolic base; Em = embolus; ET = embolic tip; FD = fertilization duct; GA = glandular appendages; ID = internal ducts; LL = lateral lobes; LS = lobal septum; MS = membranous sac; pmLL = posterior margin of lateral lobes; PP = posterior part of spermathecae; SB = sclerotised bulges; Sp = spermophor; St = subtegulum; T = tegulum; Ti = palpal tibia; vRTA = ventral branch of RTA. Scale bars: 0.5 mm (**A–F**).

**Figure 8.**
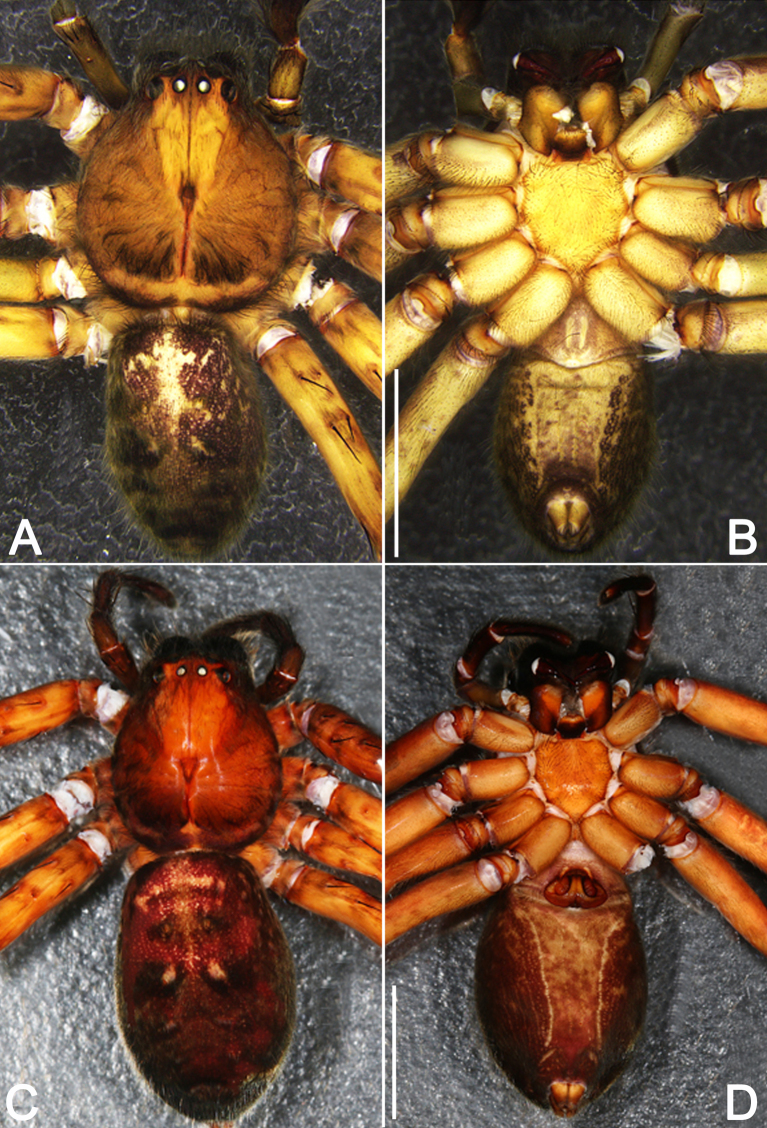
*Sinopoda tengchongensis*, materials from Honghe, male habitus (**A, B**) and female habitus (**C, D**). **A, C**. Dorsal; **B, D**. Ventral. Scale bars: 5 mm (**A–D**).

See Fu and Zhu (2008) and Grall and Jäger (2020). Male palp as in Fig. 7A–D, epigyne as in Fig. 7E, F, habitus as in Fig. 8A–D.

##### Distribution.

China (Yunnan, Hunan, Hainan), Thailand (Phrae). Distribution record in Honghe as in Fig. 1B

#### Sinopoda tumefacta

Taxon classificationAnimaliaAraneaeSparassidae

Zhong, Jäger, Chen & Liu, 2019

A30E4E6C-0053-5132-9712-33D709280FAE

Figs 1, 9, 10

Sinopoda tumefacta Zhong et al. 2019: 69, figs 53A–E, 54A–F, 55A–D (♂♀); Zhang et al. 2021: 11, fig. 5C, G, I, J (♂♀); Zhang et al. 2024: S13, figs A13E, F, A14A, B (♂♀).

##### Material examined.

***Holotype*** ♂ (CBEE), China: • Yunnan Pro.: Honghe Hani and Yi Autonomous Prefecture, Gejiu City, Pingbian Co., Daweishan Mt., 22.32°N, 103.70°E, c. 2060 m, by hand, 27 X 2015, Y. Zhong & Y. Zhu leg; ***Paratypes***: 10♂, 12♀ (CBEE), with same data as holotype; 2♂, 1♀ (CBEE), Honghe Hani and Yi Autonomous Prefecture, Gejiu City, Laoyinshan Mt., 23.36°N, 103.16°E, c. 1955 m, 29 X 2015, Y. Zhong & Y. Zhu leg.

##### Diagnosis and description.

**Figure 9.**
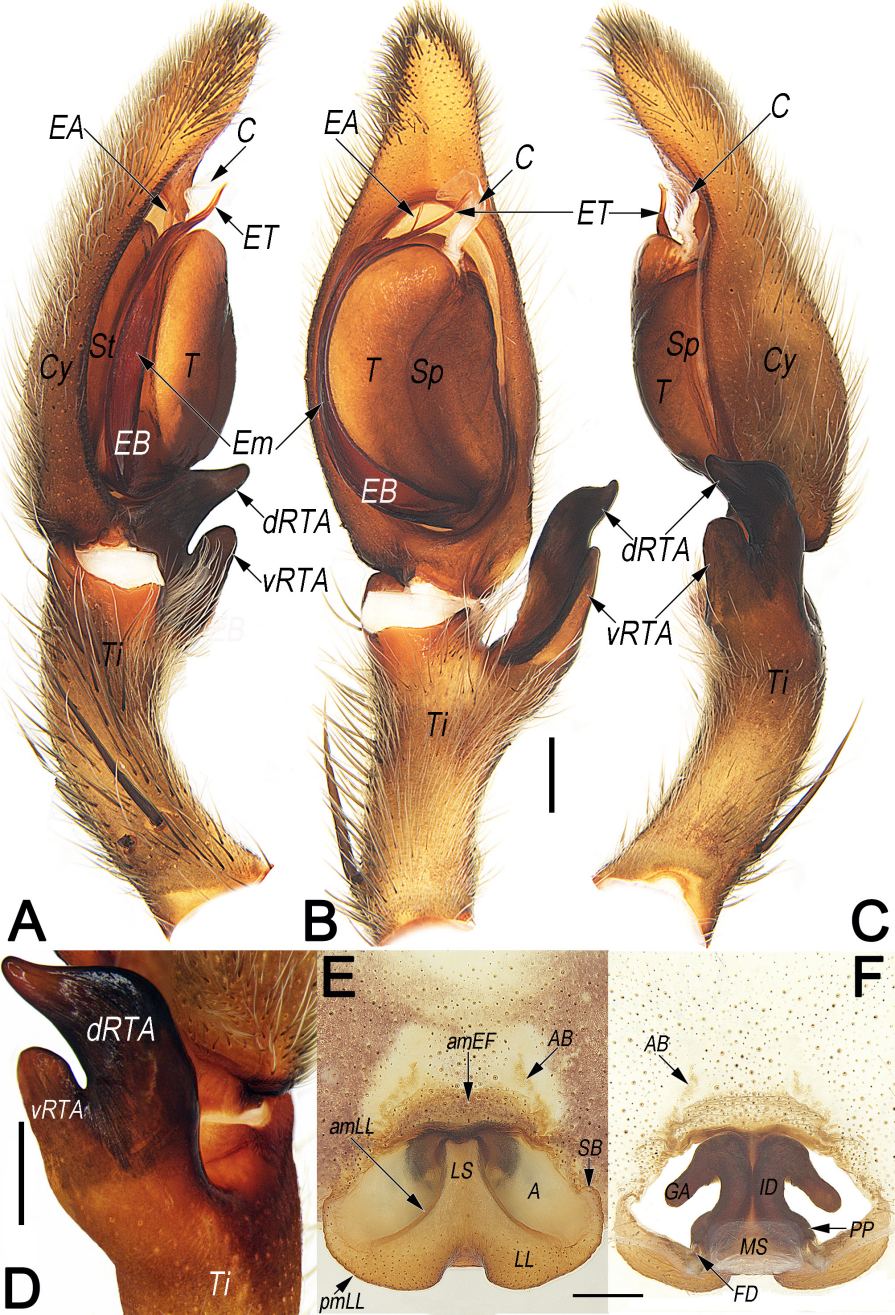
*Sinopoda tumefacta*, holotype male and paratype female from Honghe, male palp (**A–C**), palpal tibia (**D**) and epigyne (**E, F**). **A**. Prolateral; **B, E**. Ventral; **C**. Retrolateral; **D**. Dorsoretrolateral; **F**. Dorsal. Abbreviations: A = atrium; AB = anterior band; amEF = anterior margin of epigynal field; amLL = anterior margin of lateral lobes; C = conductor; Cy = cymbium; dRTA = dorsal branch of RTA; EA = embolic apophysis; EB = embolic base; Em = embolus; ET = embolic tip; FD = fertilization duct; GA = glandular appendages; ID = internal ducts; LL = lateral lobes; LS = lobal septum; MS = membranous sac; pmLL = posterior margin of lateral lobes; PP = posterior part of spermathecae; SB = sclerotised bulges; Sp = spermophor; St = subtegulum; T = tegulum; Ti = palpal tibia; vRTA = ventral branch of RTA. Scale bars: 0.5 mm (**A–F**).

**Figure 10.**
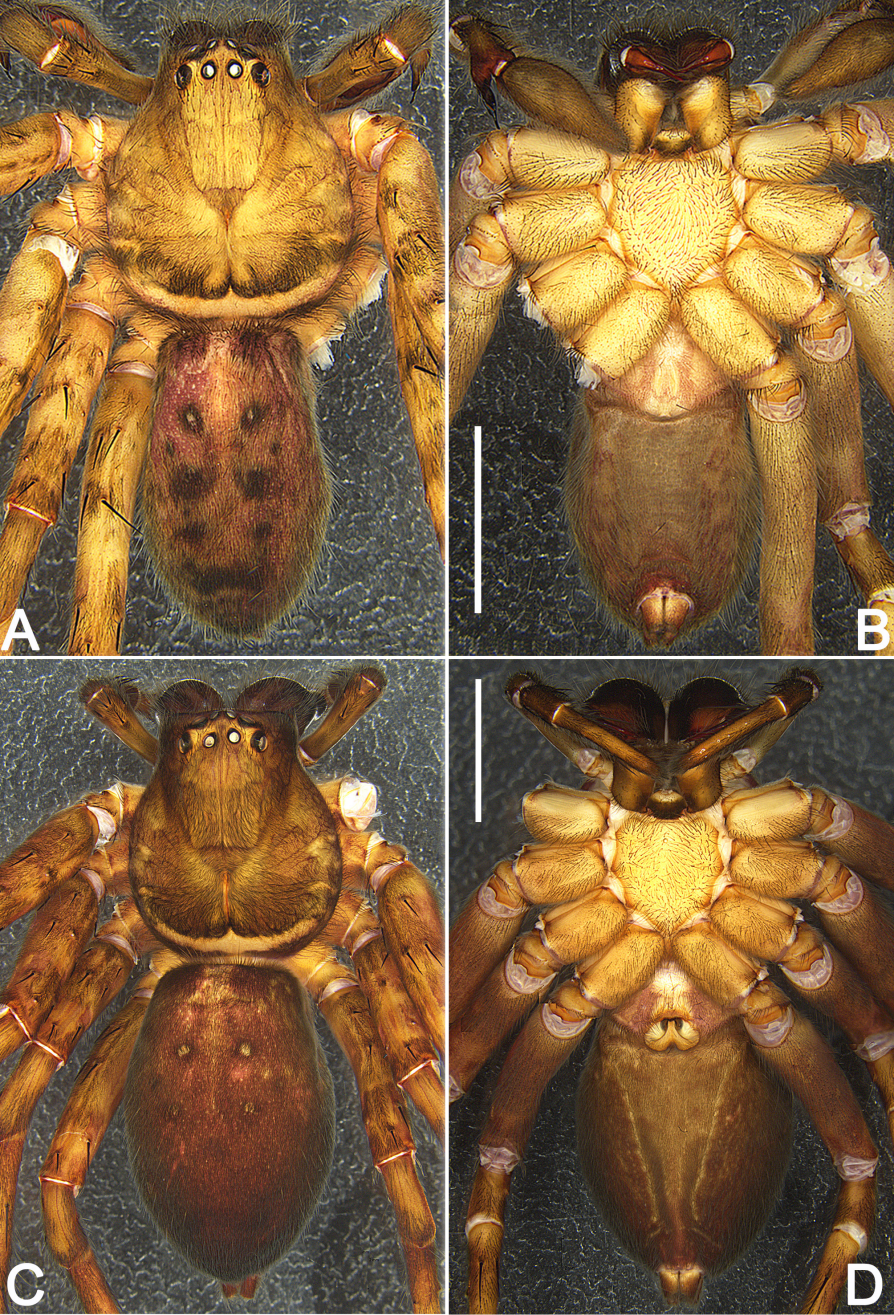
*Sinopoda tumefacta*, holotype male and paratype female from Honghe, male habitus (**A, B**) and female habitus (**C, D**). **A, C**. Dorsal; **B, D**. Ventral. Scale bars: 5 mm (**A–D**).

See Zhong et al. (2019). Male palp as in Fig. 9A–D, epigyne as in Fig. 9E, F, habitus as in Fig. 10A–D.

##### Distribution.

China (Yunnan, distribution records in Honghe as in Fig. 1B).

## Supplementary Material

XML Treatment for Sinopoda

XML Treatment for Sinopoda honghe

XML Treatment for Sinopoda kuan

XML Treatment for Sinopoda tengchongensis

XML Treatment for Sinopoda tumefacta

## Additional information

### Artificial Intelligence (AI) use

The authors accept full responsibility for the content of the manuscript, including the disclosure of any use of AI.

No AI tools were used in the preparation of this manuscript.

### Author ORCIDs

C. Zhang https://orcid.org/0009-0008-1258-5727

Y. Xing https://orcid.org/0000-0002-6278-9778

Y. Zhong https://orcid.org/0000-0002-0517-4582

H. Yu https://orcid.org/0000-0002-9113-2425

### Data availability

All of the data that support the findings of this study are available in the main text.
